# Biogeographical distribution analysis of hydrocarbon degrading and biosurfactant producing genes suggests that near-equatorial biomes have higher abundance of genes with potential for bioremediation

**DOI:** 10.1186/s12866-017-1077-4

**Published:** 2017-07-27

**Authors:** Jorge S. Oliveira, Wydemberg J. Araújo, Ricardo M. Figueiredo, Rita C. B. Silva-Portela, Alaine de Brito Guerra, Sinara Carla da Silva Araújo, Carolina Minnicelli, Aline Cardoso Carlos, Ana Tereza Ribeiro de Vasconcelos, Ana Teresa Freitas, Lucymara F. Agnez-Lima

**Affiliations:** 1 Laboratório de Biologia Molecular e Genômica, Departamento de Biologia Celular e Genética, Centro de Biociências, Universidade Federal do Rio Grande do Norte, Natal, RN Brazil; 2 INESC-ID/IST Instituto de Engenharia de Sistemas e Computadores/Instituto Superior Técnico, Universidade de Lisboa, Rua Alves Redol, 9, 1000-029 Lisbon, Portugal; 3 Laboratório de Bioinformática, Laboratório Nacional de Computação Científica, Petrópolis, RJ Brazil

**Keywords:** Hydrocarbon degradation, Biosurfactants, Environmental microbiology, Metagenomics, Metagenomics bioinformatics pipeline, Geographical ecology, Microbiome data analysis

## Abstract

**Background:**

Bacterial and Archaeal communities have a complex, symbiotic role in crude oil bioremediation. Their biosurfactants and degradation enzymes have been in the spotlight, mainly due to the awareness of ecosystem pollution caused by crude oil accidents and their use. Initially, the scientific community studied the role of individual microbial species by characterizing and optimizing their biosurfactant and oil degradation genes, studying their individual distribution. However, with the advances in genomics, in particular with the use of New-Generation-Sequencing and Metagenomics, it is now possible to have a macro view of the complex pathways related to the symbiotic degradation of hydrocarbons and surfactant production. It is now possible, although more challenging, to obtain the DNA information of an entire microbial community before automatically characterizing it. By characterizing and understanding the interconnected role of microorganisms and the role of degradation and biosurfactant genes in an ecosystem, it becomes possible to develop new biotechnological approaches for bioremediation use. This paper analyzes 46 different metagenome samples, spanning 20 biomes from different geographies obtained from different research projects.

**Results:**

A metagenomics bioinformatics pipeline, focused on the biodegradation and biosurfactant-production pathways, genes and organisms, was applied. Our main results show that: (1) surfactation and degradation are correlated events, and therefore should be studied together; (2) terrestrial biomes present more degradation genes, especially cyclic compounds, and less surfactation genes, when compared to water biomes; and (3) latitude has a significant influence on the diversity of genes involved in biodegradation and biosurfactant production. This suggests that microbiomes found near the equator are richer in genes that have a role in these processes and thus have a higher biotechnological potential.

**Conclusion:**

In this work we have focused on the biogeographical distribution of hydrocarbon degrading and biosurfactant producing genes. Our principle results can be seen as an important step forward in the application of bioremediation techniques, by considering the biostimulation, optimization or manipulation of a starting microbial consortia from the areas with higher degradation and biosurfactant producing genetic diversity.

**Electronic supplementary material:**

The online version of this article (doi:10.1186/s12866-017-1077-4) contains supplementary material, which is available to authorized users.

## Background

Studies evaluating the biogeographical influence in the diversity and/or abundance of alkane degradation and biosurfactant production genes may guide the creation of new industrial and biotechnological processes. These include bioremediation and biostimulation strategies that are important for preservation and environment planning [1, 2]. Although biogeographical studies of hydrocarbon degradation genes predominate in the literature [2], there is a relative lack of knowledge about the distribution of bacteria producing biosurfactants in the environment [3].

The synergic effects of biosurfactants on solubility, sorption and biodegradation of hydrophobic organic contaminants are known as they play an important role during biodegradation processes [4]. Biosurfactants can be synthesized by a myriad of microorganisms, which is influenced by the composition of the medium and environmental conditions [4]. However, because most studies of geographic distribution of bacteria oil-degrading genes in environments rely on the analysis of biomes that have been contaminated or enriched with crude oil, the understanding of the origin, abundance and natural role of degradation and surfactant genes on an ecosystem [3, 5, 6] has been hampered.

International microbial surveys [7–10] are good examples of large-scale coordinated efforts to explore soil and water taxonomic and functional diversity. In general, the generated datasets are available in public repositories like Sequence Read Archive (SRA). These datasets, combined with the appropriate computational pipelines, can reveal correlations between ecology and geography, based on taxonomic and functional characteristics of the biomes.

Metagenomic analysis software packages, like MG-RAST [11], MEGAN [12] and KRAKEN [13] include solutions for taxonomic, functional and comparative analyses. With these tools, metagenomic datasets are combined with global databases, which with the constantly growing size of these datasets, produces large and complex outputs that usually take several days to be analyzed. Other tools like MetAmos [14] work in a modular manner, allowing workflow customization and promise to reduce assembly errors and computational cost. However, its flexibility and modular construction makes the computational installation process time and space consuming.

Moreover, we have reached a state where the massive size of available data does not allow the use of classic brute-force bioinformatics approaches. It is thus clear that the use of domain specific studies and databases is essential to focus on a specific research scope and reduce the computational effort. In functional databases like KEGG, there are examples such as the ontology of degradation genes grouped with the beta-oxidation in the lipid metabolism pathway, or the synthesis of biosurfactants together with antibiotics in the nonribosomal peptide synthesis pathway, that make research on degradation, or surfactants individually much more difficult. To overcome this limitation, domain-specific databases, like BioSurfDB [15], reorganize the functional ontologies, thus allowing the focus, on biosurfactants and biodegradation. This domain-specific database also combines a set of tailored tools to enable efficient specific metagenomic analysis. The main goal of this tool is to support the identification of patterns of taxonomic and functional diversity of microbial communities and the identification of genes involved in the degradation of hydrocarbons and biosurfactants production.

In this research, we analyzed 46 public metagenomes, from 20 different biomes, water and terrestrial, to increase our understanding of the biogeographical distribution of biodegradation and biosurfactant-production genes. Additionally, a metagenomics pipeline that relies on BioSurfDB, to effectively and efficiently process a large amount of data, was developed and optimized.

## Methods

All the computational processing was performed in a AMD server running Slackware version 14 in 64bits, with 64 CPUs and 258GB of RAM.

**Fig. 1:**
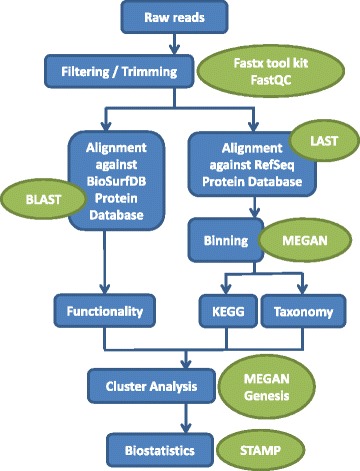
Computational pipeline for taxonomic and functional analysis. The main processing steps are in *blue* and the software used is highlighted in *green*

Metagenome sequences were downloaded from the SRA at NCBI website, the Metagenomic samples detailed information on SRA project and Run are available in Suplementary Material (Additional file 1: Table S1). The Metagenomes Summary table (Additional file 2: Table S2), summarizes the information regarding both soil and water metagenomes. Whenever possible, several samples from each biome were selected. There were a total of 71 DNA-seq metagenomes with a heterogeneous set of possible environmental samples with worldwide representation. Sample Geography figure (Additional file 3: Figure S1) presents a geographic distribution of the metagenomes that have been analyzed. The pipeline presented in Fig. 1 was used to get a macro view of the taxonomic and functional differences between the metagenomes.

### Filtering/Trimming

A filtering/trimming procedure was applied to all the metagenomes presenting low quality parameters in the FASTQC (http://www.bioinformatics.babraham.ac.uk/projects/fastqc) report. Based on the generated quality report, the trimming of k-mer contaminated and heterogeneous GC-content areas was performed using *Fastx toolkit trimmer*. Also *fastq_quality_filter* from the same toolkit was used to assure a minimum Phred-Score of 20 for at least 90% of the reads. This procedure revealed to be an iterative and supervised-dependent process, as it had to be repeated for some samples until the FASTQC reports showed acceptable quality. The final number of sequences was also analyzed and the metagenomes with less than 100.000 sequences were discarded. It was decided to use a more conservative approach, by using less samples but with higher quality per sample.

### Alignment

After the quality assessment, two parallel alignment steps were performed: (i) an alignment against BioSurfDB, a domain-based database, and (ii) an alignment against the RefSeq, a generic sequence database [16].

At this stage we should stress that the alignment was carried out using all the reads in the datasets and no assembling was performed to obtain contigs. This decision was significant and was based on the following observed during a preliminary study that had evaluated the impact of using contigs when abundance analysis is performed:

1. if the goal is to compare gene abundances between metagenomes, the use of contigs instead of reads will significantly reduce the abundance of information leading to inaccurate results;
2. in metagenomics, the organism diversity is so high that it is very difficult for assemblers to distinguish a repeated read from a homolog one, thus masking the real number of organisms present in the datasets;
3. when dealing with a large amount of heterogeneous sequencing data, average read length, coverage or quality a consistently high quality assembly step might not be possible because of the sequencing technology used.

### RefSeq

RefSeq is a non-redundant database integrating sequences from many sources. The full set of non-redundant protein sequences (9.5 GBs) was downloaded. The selected sequence alignment program was LAST [17], an aligner optimized for repeat-rich datasets that performs much faster than the traditional BLAST [18]. This algorithm is very useful in situations where the size of the data hampers the alignment. Each metagenome was aligned to the RefSeq database using the default parameters for the LAST aligner. Taxonomic and Functional binning was performed by MEGAN (version 5) using its respective RefSeq and KEGG maps databases.

### BioSurfDB

BioSurfDB is a curated information system with a focus on biodegradation and biosurfactant production organisms. It was developed to support research in the bioremediation field. This information system includes tools for the alignment of metagenomes against a number of genomic or protein sequences. One sample of each group of metagenomes, in a total of 46 samples, was uploaded to the BioSurfDB system and the BLASTx tool. Nucleotide query versus protein database with an E-value of 1e^−4^ was used for sequence alignment. Currently, the BioSurfDB database includes 3956 protein sequences from different pathways. The list of pathways available in BioSurfDB at the time of this study is shown in the BioSurfDB Pathways table (Additional file 4: Table S3). Following the alignment, the BioSurfDB system automatically performs taxonomic and functional binning. However, as BioSurfDB is a domain-specific database, its taxonomic prediction might be biased and therefore, we decided not to use it for taxonomic classification.

### Cluster analysis

Alignment results from all the analyzed metagenomes, from both BioSurfDB and RefSeq analysis, were uploaded to MEGAN to compute UPGMA trees and PCoA (Principal Coordinates Analysis).

The metagenomics computational pipeline used includes scripts that cross the BLASTx results and the database tree, creating hit-count tables for taxonomy, proteins and metabolic pathways. These pathway tables were uploaded to Genesis [19], where normalization was applied, followed by the calculation of hierarchical clustering for both metagenomes and pathways, using a complete link approach.

### Statistics

Results from the BLAST alignment using the BioSurfDB as database were grouped in a metadata file, according to the functional clusters obtained in the previous step. These tables were uploaded to STAMP [20] to perform the statistical tests between metagenomes and to Graphpad Prism to test the correlation between the surfactant production and hydrocarbon degradation.

To calculate the correlation coefficient between the diversity, i.e., the number of different blast alignments mapped, of biosurfactant and degradation genes in the environment, a Pearson parametric test was used, with a confidence interval of 0.95 and a *P*-value <0.0001.

A preliminary data analysis, automatically performed by STAMP, decides which is the best statistical test to be performed. A two-sided Welch’s t-test with a confidence interval of 0.95 and Benjamini-Hochberg multiple test correction was performed to identify significant differences between groups. Two filters were used: a minimal q-value of 0.05 and a minimum difference of proportions of 1 (program defaults).

## Results

### Quality assessment

From the initial dataset of 71 metagenomics samples, 24 samples were discarded by failing the quality assessment, and 46 samples from the several biomes, shown in Table 1 were used for further analysis. At this stage of the data analysis, it was not possible to guarantee a uniform number of samples per biome, because for many of the projects the samples were not of acceptable quality.

**Table 1:** Analyzed Biomes, classified by soil or water type, with information about the region, number of reads, average read length, sequencing technology used and sequencing project SRA code and link

	Regions	Number of reads	Read length (bp)	Seq. Tech.	SRA Link
Soil					
Tundra	Siberia & Canada	1.31E + 07	183.5	Illumina	SRP047512
Temp. Woodland	Australia	1.23E + 07	290	Illumina	ERP008551
Arid Grassland	Australia	1.92E + 07	299	Illumina	ERP008551
Saline Desert	India	2.07E + 06	124	Ion	SRP041239
Atlantic Forest	Brazil	9.62E + 04	380	Illumina	SRP004544
Tropical Forest	French Guiana	4.04E + 05	384	454	ERP002426
Temp. Coniferous Forest	Canada	2.18E + 07	136	Illumina	ERP009498
Mangrove	Brazil	5.26E + 05	418	454	SRP004544
Caatinga	Brazil	2.31E + 05	426	454	SRP004544
Paddy Soil	China	2.16E + 06	190	Illumina	SRP039858
Temp. Plantation Soil	Australia	3.32E + 07	299	Illumina	ERP008551
Grassland Soil	Oklahoma	9.43E + 06	169	lllumina	SRP029969
Terrestrial Subsurface	South Africa	1.11E + 07	186	Illumina	SRP049336
Water					
Sea Water	North Pacific	2.67E + 07	187	Illumina	ERP003628
Sea Water	South Pacific	2.66E + 07	188	Illumina	ERP003628
Sea Water	Indian Ocean	1.63E + 07	185	Illumina	ERP001736
South Atlantic	Brazil	2.46E + 07	184	Illumina	ERP003708
North Atlantic	Iceland	8.39E + 05	460	Illumina	ERP009703
North Atlantic	Portugal	3.32E + 06	293	Illumina	ERP009703
River Plume	Amazon	5.23E + 06	286	Illumina	SRP039390
Adriatic / Ionian Sea	Mediterranean	9.62E + 07	193	Illumina	ERP003628
River Estuary	Brazil	1.00E + 05	438	454	SRP004544

All data and metadata can be retrieved from the link provided

### Taxonomy annotation using RefSeq

**Fig. 2:**
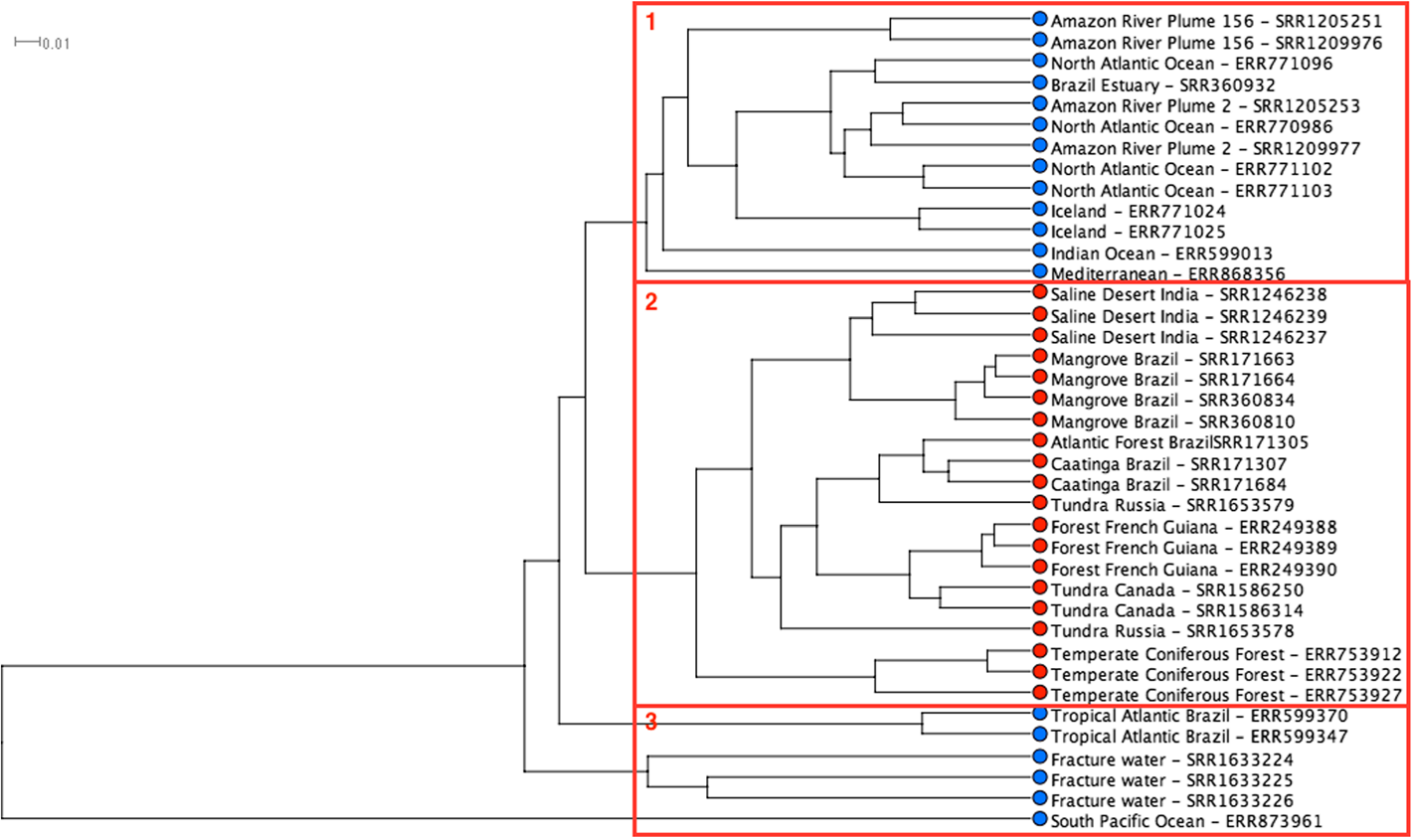
UPGMA Tree computed by MEGAN with RefSeq data. The distance between the clusters is based on pairwise distance among taxa. The soil samples are represented with a *red dot* and the water samples with *blue*. The *red squares* show the proposed cluster division

For the 46 samples, the metagenomes annotations were obtained by using the alignment program LAST to compare the metagenomic sequences with the RefSeq protein database. The obtained results were grouped using a hierarchical clustering algorithm available in MEGAN. Unfortunately, due to the large size of the metagenomes, our server could not process 8 of these samples in MEGAN. Therefore, and solely in the hierarchical cluster step, only 39 samples, corresponding to 17 biomes were analyzed. The results in Fig. 2 show the formation of distinct taxonomic clusters. From the dendrogram analysis we have considered three different clusters. In cluster 1, it is possible to see water metagenomes, mainly samples from the Atlantic and Pacific oceans and grouped into distinct cluster extensions. The second cluster includes only terrestrial metagenomes. However, it is possible to verify the grouping of terrestrial metagenomes by similar climatic regions. The third cluster is also formed by water metagenomes, but from tropical regions.

These results validate the samples for consistency, as the samples from the same metagenomes are in the same clusters. Based on this clustering result, we decided to use just one sample dataset as a representative of each metagenome for further analysis. Consequently, it was possible to optimize the use of computational resources.

Furthermore, we computed a rarefaction curve in the MEGAN tool, to assure that the metagenomic datasets included a significant number of reads to cover most taxons. As seen in the Rarefaction Curve figure (Additional file 5: Figure S2), the number of leaves in taxonomy reaches a plateau in all samples and this confirms the acceptable sample size of the data under analysis.

### BioSurfDB cluster analysis

**Fig. 3:**
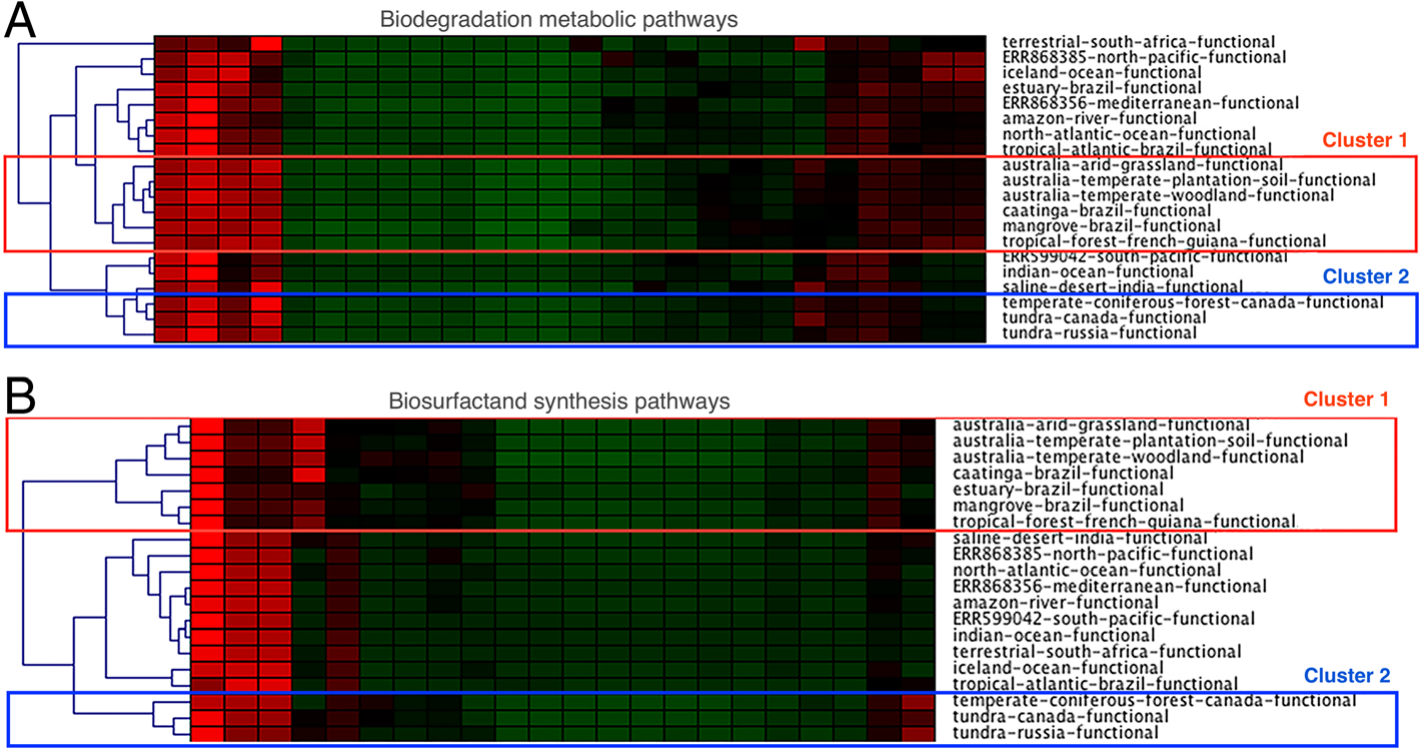
Hierarchical clusters obtained from the BioSurfDB functional data through Genesis software, for degradation (**a**) and biosurfactants (**b**). Inside the *red borders* the “equatorial region clusters” can be seen whilst inside the *blue borders* are the “cold region clusters”. Each column represents a specific pathway and the colour schema for their relative abundances is *green* for low and *red* for a high number of blast hits

Using the 46 samples, a functional clustering was carried out examining the data obtained from a BLAST compared with the databases included in the BioSurfDB information system using the Genesis software tool. Figure 3 shows the resulting hierarchical clusters when only the degradation genes are considered, see Fig. 3a, and when considering only the biosurfactants production genes, see Fig. 3b. K-means clustering was also used and revealed clusters like those obtained by the hierarchical clustering algorithm.

These results highlight two important clusters: (1) a cluster of tropical or near-equatorial terrestrial metagenomes (represented by the red square in Fig. 3 (a) and (b)) that show the highest values of reads mapping both for degradation and biosurfactant genes, showing the similarity of the microorganism communities; and (2) metagenomes from cold regions in Russia and Canada (blue square) that have a low abundance of microorganisms involved in the degradation of biosurfactant production processes.

**Fig. 4:**
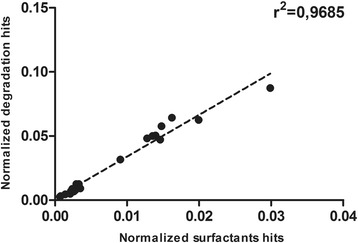
Linear correlation between biosurfactant and degradation gene diversity

A global analysis of the 46 samples resulted in an important correlation between the diversity of biosurfactant genes when compared with the existence of degradation genes in the environment (Fig. 4). The parametric Pearson-correlation test showed a positive linear correlation, with an R^2^ of 0.9, suggesting that both biosurfactant and biodegradation genetic diversity are related. This and the observations presented by the *BioSurfDB Cluster Analysis* underlined the importance of analyzing both biosurfactant and biodegradation genes at the same time.

### Statistics

**Fig. 5:**
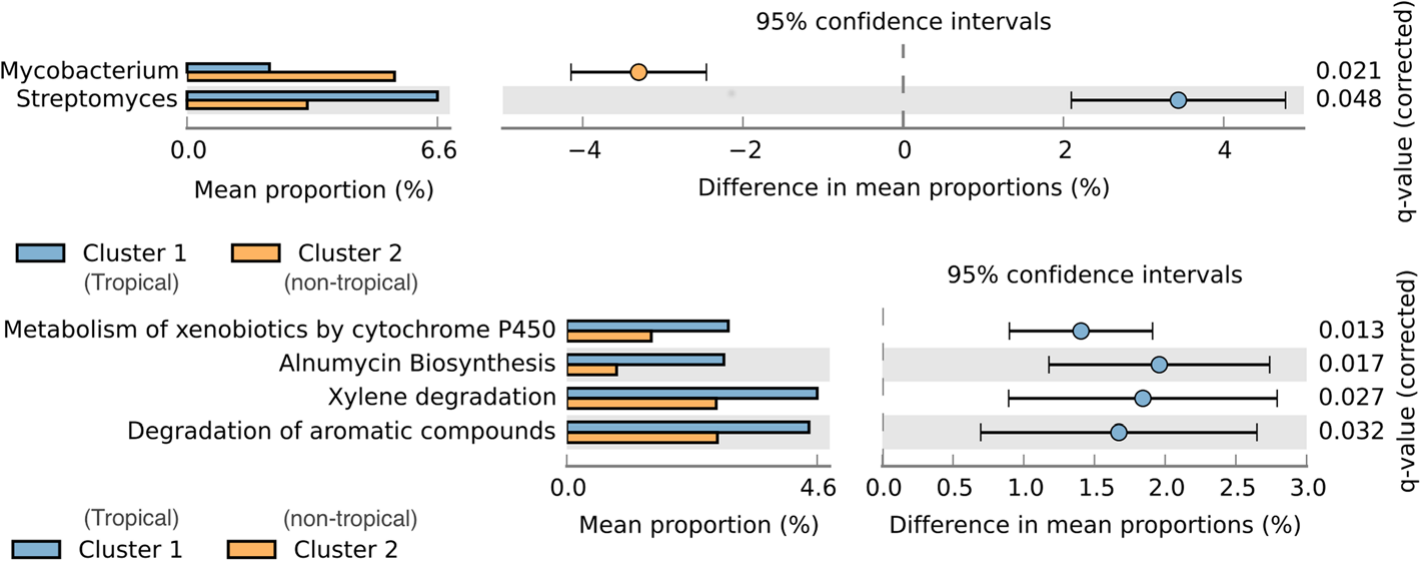
Significant taxonomic (*above*) and functional (*below*) differences between Cluster 1 (tropical) and Cluster 2 (non-tropical). Computed in STAMP tool

According to the results presented in Fig. 5, from the comparison of the two most distant clusters: *Cluster 2,* non-tropical metagenomes and *Cluster 1*, tropical metagenomes, it is possible to identify significant differences of more than 3% in the abundance of the microorganisms’ genus. The abundance of *Mycobacterium* is significantly higher in *Cluster 2*, while Streptomyces is more abundant in *Cluster 1*.

Regarding the comparison of functional data, see Fig. 5b, the results demonstrate a significant prevalence of degradation of aromatic hydrocarbon genes in Cluster 1, composed of tropical metagenomes. These genes are associated with xylene and aromatic degradation, the metabolism of xenobiotics by cytochrome P450 and alnumycin biosynthesis.

**Fig. 6:**
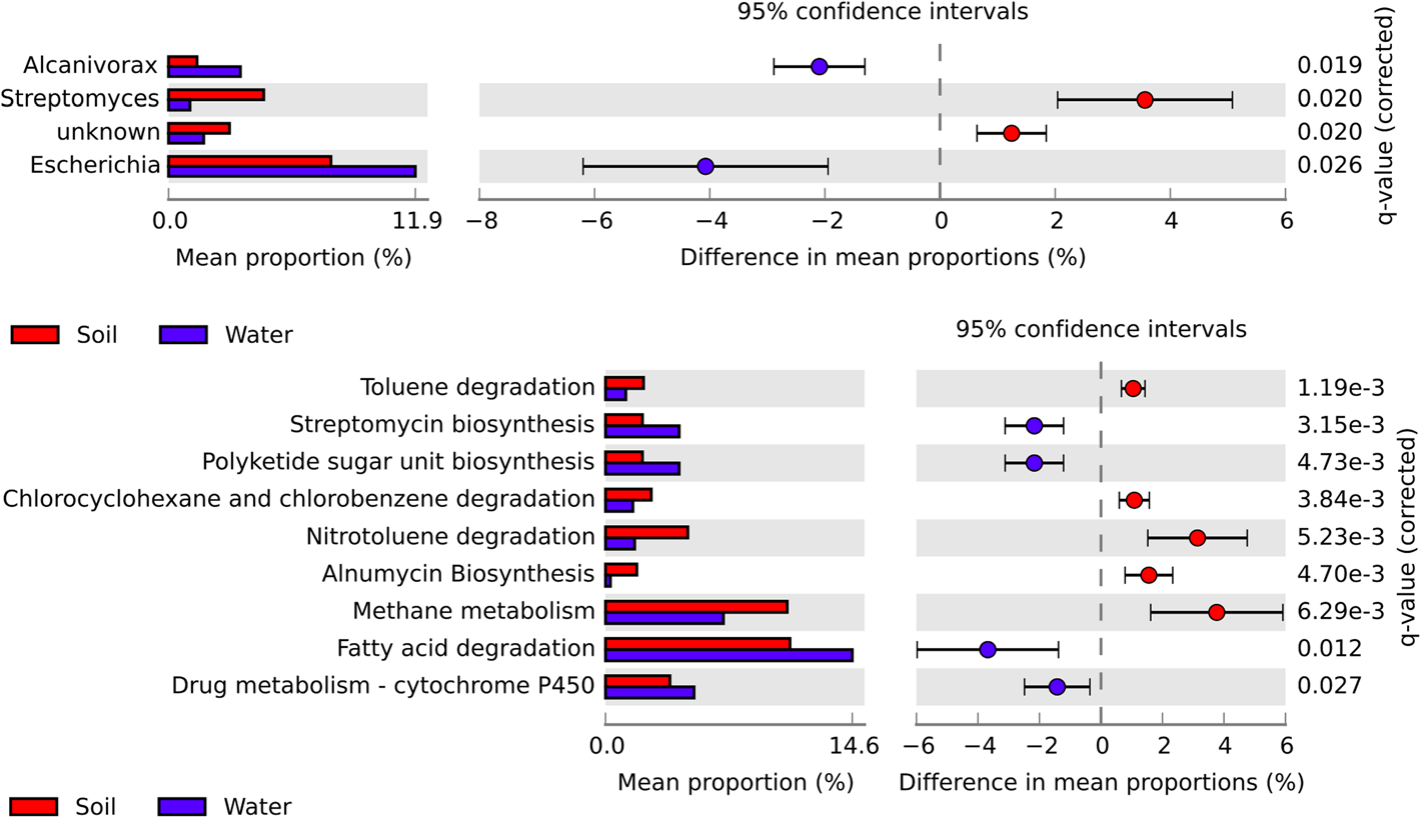
Significant taxonomic (*above*) and functional (*below*) differences between soil and water clusters. Computed in STAMP tool

From a different perspective, the soil and water metagenomes were also compared (Fig. 6). The *Alcanivorax* and *Escherichia* genera are more abundant in water metagenomes, while *Streptomyces* is more abundant in terrestrial metagenomes.

Functional comparison of terrestrial and water samples revealed that some cyclic hydrocarbon degradation pathways, namely toluene, chlorocyclohexane, chlorobenzene and nitrotoluene degradation are significantly more abundant in terrestrial metagenomes, while linear hydrocarbon degradation pathways, as alkane degradation and cytochrome P450 metabolism are significantly more abundant in water ecosystems. In addition, streptomycin and polyketides biosynthesis pathways are more representative of the water biomes, while Alnumycin biosynthesis is more abundant in terrestrial biomes. Methane metabolism is also significantly higher in terrestrial biomes.

## Discussion

In this article we have focused on the biogeographical distribution of hydrocarbon degrading and biosurfactant producing genetic diversity, in the environment.

### Taxonomic analysis using RefSeq

Using Refseq databases, the formation of clusters from water and terrestrial metagenomes are in accordance with previous studies suggesting that the principle factor influencing the microbiota is if the substrate is terrestrial or water [21]. On a second level, metagenomes subjected to similar abiotic and biotic conditions such as sunlight, temperature, oxygen supply, osmotic and redox potential, pH and nutrient supply should have a similar bacterial community in their environments [22]. Therefore, these factors possibly determine the formation of the clusters observed in this paper.

### Functional analysis using BioSurfDB

In the functional analysis performed using BioSurfDB, we analyzed all the available genes involved with the hydrocarbon degradation pathways along with the genes of the biosurfactant synthesis (Cluster 1 in Fig. 3). One of the main reasons for this analysis was the fact that biodegradation is favored by the biosurfactant miscibility effect on hydrophobic material in order to assure its biodisponibility for bacteria.

Temperature is another factor that directly affects hydrocarbon biodegradation [23, 24]. Low temperatures are an important limitation to hydrocarbon biodegradation because they generate suboptimal environmental conditions for biodegradation such as increased viscosity, retarded volatilization of short-chain alkanes that are <C10, insolubility of long-chain alkanes, limited availability of water and nutrients; specifically, nitrogen, and extremes in pH and salinity [25]. In contrast, higher temperatures increase the rates of hydrocarbon metabolism to a maximum, typically in the range of 30 to 40 °C [23]. Moreover, in tropical areas there are high incidences of light and high average temperatures that favor photoautotrophic organisms, such as plants, algae and cyanobacteria, which can naturally synthesize linear or aromatic hydrocarbons [26–28]. Therefore, the higher occurrence of degrading organisms and producers of biosurfactants in tropical areas see cluster 1 in Fig. 3) is probably favored by the documented higher bioavailability of hydrocarbons in these regions when compared with cold regions, see cluster 2 in Fig. 3 [4, 29, 30].

### Correlation between biosurfactant production and biodegradation

The strong correlation (0.9) between degradation genes and genes involved in the biosynthesis of biosurfactants, observed in this study, reinforces the need for more research on biogeography distribution of both degradation and biosurfactants synthesis genes, to increase our understanding of their integrated action in the environment. This evidence is an important contribution to this knowledge, as most of the existing biogeographical studies on degradation and surfactant gene abundance analyze those pathways separately [1–3].

### Statistical comparisons

#### Tropical vs. Non-tropical regions

*Streptomyces* and *Mycobacterium* are the most represented genus in tropical areas (Cluster 1 in Fig. 5) and non-tropical (Cluster 2 in Fig. 5), respectively. Both genera are described as capable of degrading hydrocarbons and produce biosurfactants [4]. In fact, hydrocarbon-degrading microorganisms are ubiquitous in several ecosystems, although they constitute less than 0.1% of the microbial community. However, in oil-polluted environments, they can represent up to 100% of the viable microorganisms [31]. Therefore, when we analyze the abundance of these genes in contaminated environments we are not only observing the natural dynamic or abundance of the bacterial community.

In this study, *Mycobacterium*, included in the *Actinobacteria* phylum, was the most representative genera in non-tropical (Cluster 2 in Fig. 5). Similarly, the first metagenomic analysis of permafrost samples showed Actinobacteria as a dominant phylum in accordance with the community composition reported from other polar soils [32]. Biofilm formation has been suggested to optimize the bioavailability of the substrate necessary for the growth of *Mycobacterium* under low concentrations of anthracene (PAH) [33]. However, biosurfactant production was not observed for *Mycobacterium* [33], which can explain the low abundance of surfactants in our results.

### Soil vs. Water metagenomes

In this study, *Escherichia* and *Alcanivorax* genus were predominant in water metagenomes while *Streptomyces* was shown to be abundant in terrestrial metagenomes. *Escherichia* belongs to the *Enterobacteriaceae* family, which is not expected to show extracorporeal existence. However, the success of *E. coli* in the gut ecosystem, an example of a harsh environment, is thought to reflect its abilities to occupy different ecological niches. Corroborating this hypothesis, recent studies reporting the isolation of indigenous *E. coli* able to degrade hydrocarbon from contaminated soils [34, 35] showed the property of another bacterium from the *Escherichia* genus, the *E. fergusonii* KLU01, isolated from oil contaminated soil, as a hydrocarbon degrading, heavy metal tolerant and a potent producer of biosurfactant using diesel oil as the sole carbon and energy source [36]. Similarly, Sarma et al. 2004, isolated an enteric strain *Leclercia adecarboxylata* PS4040 from soil samples, collected from an oily sludge contaminated site that had had a contamination history of over 100 years, which is genotypically different from a clinical strain of *L. adecarboxylata* and showed that it can degrade other two- and three-benzene-ring PAH [37].

In water metagenomes, the *Alcanivorax* hydrocarbonoclastic genus is predominant when compared to those in soil. Despite being predominantly marine and described as almost exclusively linear alkane degrading and being up to 90% present in seawater contaminated with petroleum [38], it has also been found in some saline terrestrial environments contaminated with hydrocarbons [39]. Alkanes are open-chain hydrocarbons, which may represent up to 50% of the crude oil [40], and may also be synthesized by cyanobacteria [41], being rapidly degraded in marine environments [42]. Furthermore, the functional analysis of this study shows the predominance of the linear alkanes degradation pathway (fatty acid degradation) in water metagenomes and the predominant degradation genes of P450. This is probably due to the high incidence of the *Alcanivorax* genus that has a highly restricted genome of catabolic enzyme, since this organism uses predominantly aliphatic hydrocarbons as a source of carbon and energy and has several well-annotated genes encoding for AlkB1 and AlkB2 and Cytochrome P450 [43]. Furthermore, *Alcanivorax* and *Streptomyces*, are significantly abundant in clusters with a prevalence of genes involved in biosurfactant synthesis and hydrocarbon degradation which have also already been reported as biosurfactant producers [43–45].

Moreover, other studies noted the predominance of aromatic compound degradation genes in soil [46] when compared to alkane degradation genes AlkB. We observed the predominance of aromatic degradation genes in soil when compared with water metagenomes. This is possibly justified by the fact that polycyclic aromatic compounds are released into the atmosphere due to the use of fossil fuels and are subjected to chemical and physical degradation. Consequently, soils are the primary repository of aromatic compounds due to their capacity for retaining hydrophobic compounds [47]. *Streptomyces* are also typical soil bacteria already described as capable of utilizing PAH and petroleum as carbon and energy sources [36, 37]. Our results are in accordance with this, as they showed significant predominance of *Streptomyces* in soil metagenomes.

### Computational challenges

One of the main challenges in this research was investigating the possibility of obtaining new knowledge from the analysis of heterogeneous and publicly available metagenomics datasets. Advanced analytics, associated with high-performance computing, has made possible a more comprehensive analysis of many metagenomes. However, data integration often revealed deficiencies in data quality, e.g. inconsistency, redundancy, poor annotations and incompleteness. It was also clear that although the proposed bioinformatics pipeline could produce very interesting results, additional types of data should be considered to improve the knowledge regarding gene diversity. A more comprehensive analysis of these datasets should include DNA-Seq and RNA-Seq data to understand the ultimate activity of the identified genes.

One important result of this study is that the metagenomics data that is publicly available still needs to be improved in terms of its quality. Most of the available datasets are of poor quality, limiting the statistical significance of further analysis. In this research we have faced a 34% reduction in the size of the datasets when compared with the raw data.

## Conclusion

From our research It was possible to see that: (1) surfactation and degradation are correlated events; (2) terrestrial biomes have more degradation genes, especially cyclic compounds, and less surfactation genes when compared to water biomes; and (3) latitude has a significant influence on the diversity of genes involved in biodegradation and biosurfactant production, suggesting that microbiomes near the equator have richer genes that have a role in these processes.

This information can be used in the application of bioremediation techniques, by taking into considering the biostimulation, optimization or manipulation of microbial consortia from these areas.

## Additional files

Additional file 1: Table S1. Metagenomes Summary. Country, number of samples and sequencing technology for each biome. (DOCX 66 kb)

Additional file 2: Table S2. Samples Information. Feature, location, run and project SRA information for each sample. (DOCX 120 kb)

Additional file 3: Figure S1. Sample Geography. Geographical distribution of the metagenome samples. (DOCX 1078 kb)

Additional file 4: Table S3. BioSurfDB Pathways. Name and KEGG Map ID for Alkane biodegradation and surfactant biosynthesis pathways analyzed. (DOCX 102 kb)

Additional file 5: Figure S2. Rarefaction Curve. Rarefaction Curves performed in MEGAN. (DOCX 2185 kb)

## Abbreviations

CPU: Central processing unit
DNA: Deoxyribonucleic acid
DNA-Seq: DNA sequencing
GC: Guanine-cytosine
NCBI: National Center for Biotechnology Information
PAH: Poly-aromatic-hydrocarbons
PCoA: Principal coordinates analysis
RAM: Random-access-memory
RNA: Ribonucleic acid
RNA-Seq: RNA sequencing
UPGMA: Unweighted pair group method with arithmetic mean

## References

[1] Kurata N, Vella K, Hamilton B, Shivji M, Soloviev A, Matt S, Tartar A, Perrie W (2014). Surfactant-associated bacteria in the near-surface layer of the ocean. Sci Rep.

[2] Jan BVB, Li Z, Wouter D, Last BW (2003). Diversity of alkane hydroxylase systems in the environment. Oil Gas Sci Technol.

[3] Bodour AA, Drees KP, Maier RM (2003). Distribution of biosurfactant producing bacteria in undisturbed and contaminated arid southwestern soils. Appl Environ Microbiol.

[4] Jitendra DD, Ibrahim MB. Microbial production of surfactants and their commercial potential. Microbiol Mol Biol Rev. 1997:61(1):47–64. 10.1128/mmbr.61.1.47-64.1997 PMC232600 9106364

[5] Hassanshahian M, Zeynalipour MS, Musa FH (2014). Isolation and characterization of crude oil degrading bacteria from the Persian Gulf (Khorramshahr provenance). Mar Pollut Bull.

[6] Wallisch S, Gril T, Dong X, Welzl G, Bruns C, Heath E, Engel M, Suhadolc M, Schloter M (2014). Effects of different compost amendments on the abundance and composition of alkB harboring bacterial communities in a soil under industrial use contaminated with hydrocarbons. Front Microbiol.

[7] Gilbert JA, Jansson JK, Knight R (2014). The earth microbiome project: successes and aspirations. BMC Biol.

[8] Delmont TO, Robe P, Cecillon S, Clark IM, Constancias F, Simonet P, Hirsch PR, Vogel TM (2009). Terra genome: a consortium for the sequencing of a soil metagenome. Nat Rev Microbiol.

[9] Pylro VS, Roesch L, Ortega JM, do Amaral AM. (2014). Brazilian microbiome project: revealing the unexplored microbial diversity— challenges and prospects. Microb Ecol.

[10] Nesme J, Achouak W, Agathos SN, Bailey M, Baldrian P, Brunel D, Frostegård A, Heulin T, Jansson JK, Jurkevitch E, Kruus KL, Kowalchuk GA, Lagares A, Lappin-Scott HM, Lemanceau P, Paslier DL, Mandic-Mulec I, Murrell JC, Myrold DD, Nalin R, Nannipieri R, Neufeld JD, Gara FO, Parnell JJ, Pühler A, Pylro V, Ramos JL, Roesch LFW, Schloter M, Schleper C, Sczyrba A, Sessitsch A, Sjöling S, Sørensen J, Sørensen SJ, Tebbe CC, Topp E, Tsiamis G, JDV E, Keulen GV, Widmer F, Wagner M, Zhang T, Zhang X, Zhao L, Zhu YG, Vogel TM, Simonet P (2016). Back to the future of soil metagenomics. Front Microbiol.

[11] Meyer F, Paarmann D, D'Souza M, Olson R, Glass EM, Kubal M, Paczian T, Rodriguez A, Stevens R, Wilke A, Wilkening J, Edwards RA (2008). The metagenomics RAST server – a public resource for the automatic phylogenetic and functional analysis of metagenomes. BMC Bioinf.

[12] Huson DH, Mitra S, Ruscheweyh H, Weber N, Schuster SC (2011). Integrative analysis of environmental sequences using MEGAN4. Genome Res.

[13] Wood DE, Salzberg SL (2014). Kraken: ultrafast metagenomic sequence classification using exact alignments. Genome Biol.

[14] Treangen TJ, Koren S, Sommer DD, Liu B, Astrovskaya I, Ondov B, Aaron BO (2013). MetAMOS: a modular and open source metagenomic assembly and analysis pipeline. Genome Biol.

[15] Oliveira JS, Araújo W, Sales AIL, Guerra AB, Araújo SCS, Vasconcelos ATR, Agnez-Lima LF, Freitas AT. BioSurfDB: knowledge and algorithms to support biosurfactants and biodegradation studies. Database. 2015;2015:1–8. https://doi.org/10.1093/database/bav033. 10.1093/database/bav033 PMC4381105 25833955

[16] Tatusova T, Ciufo S, Fedorov B, O'Neill K, Tolstoy I (2014). RefSeq microbial genomes database: new representation and annotation strategy. Nucleic Acids Res.

[17] Kiełbasa SM, Wan R, Sato K, Horton P, Frith MC (2011). Adaptive seeds tame genomic sequence comparison. Genome Res.

[18] Altschul SF, Gish W, Miller W, Myers EW, Lipman DJ (1990). Basic local alignment search tool. J Mol Biol.

[19] Sturn A, Quackenbush J, Trajanoski Z (2002). Genesis: Cluster analysis of microarray data. Bioinformatics.

[20] Parks DH, Tyson GW, Hugenholtz P, Beiko RG (2014). STAMP: Statistical analysis of taxonomic and functional profiles. Bioinformatics.

[21] Jeffries TC, Seymour JR, Gilbert JA, Dinsdale EA, Newton K, Leterme SSC, Roudnew B, Smith RJ, Seuront L, Mitchell JG (2011). Substrate type determines metagenomic profiles from diverse chemical habitats. PLoS One.

[22] Standing D, Killham K. 2006. Modern soil microbiology, 2nd Edition. Chapter 1, International standard book number-13: 978-1-4200-1520-1.

[23] Bossert I, Bartha R, Atlas RM (1984). The fate of petroleum in soil ecosystems, p. 434-476. Petroleum microbiology.

[24] Davis SJ, Gibbs CF (1975). The effect of weathering on crude oil residue exposed at sea. Water Res.

[25] Margesin R (2000). Potential of cold-adapted microorganisms for bioremediation of oil-polluted Alpine soils. Int Biodeterior Biodegrad.

[26] Pattanaik B, Lindberg P (2015). Terpenoids and their biosynthesis in cyanobacteria. Life.

[27] Winters K, Parker PL, van Baalen C (1969). Hydrocarbons of blue-green algae: geochemical significance. Science.

[28] Timmis KN, McGenity TJ, van der Meer JR, de Lorenzo V (2010). Handbook of hydrocarbon and lipid microbiology.

[29] Leahy JG, Colwell RR (1990). Microbial Degradation of Hydrocarbons in the Environment. Microbiol Rev.

[30] Eliora ZR, Eugene R (2001). Natural roles of biosurfactants. Environ Microbiol.

[31] Atlas RM (1981). Microbial degradation of petroleum hydrocarbons: an environmental perspective. Microbiol Rev.

[32] Barabas G, Vargha G, Szab IM, Penyige A, Damjanovich S, Szöllösi J, Matk J, Hirano T, Matyus A, Szab I (2001). n-Alkane uptake and utilisation by Streptomyces strains. Antonie Van Leeuwenhoek.

[33] Wick LY, de Munain AR, Springael D, Harms H (2002). Responses of *Mycobacterium sp.* LB501T to the low bioavailability of solid anthracene. Appl Microbiol Biotechnol.

[34] Yergeau E, Hogues H, Whyte LG, Greer CW (2010). The functional potential of high Arctic permafrost revealed by metagenomic sequencing, qPCR and microarray analyses. ISME J.

[35] Ferradji FZ, Fodil D, Mnif S, Eddouaouda K, Badis A, Rebbani S, Sayadi S (2014). Naphthalene and crude oil degradation by biosurfactant producing *Streptomyces spp.* isolated from Mitidja plain soil (North of Algeria). Int Biodeterior Biodegrad.

[36] Shekhar SK, Godheja J, Modi DR (2014). Hydrocarbon bioremediation efficiency by five indigenous isolated from contaminated soils. Int J Curr Microbiol Appl Sci.

[37] Sriram MI, Kalishwaralal K, Deepak V, Gracerosepat R, Srisakthi K, Gurunathan S (2011). Biofilm inhibition and antimicrobial action of lipopeptide biosurfactant produced by heavy metal tolerant strain *Bacillus cereus* NK1. Colloids Surf B: Biointerfaces.

[38] Sarma PM, Bhattacharya D, Krishnan S, Lal B (2004). Degradation of polycyclic aromatic hydrocarbons by a newly discovered enteric bacterium, *Leclercia adecarboxylata*. Appl Environ Microbiol.

[39] Harayama S, Kishira H, Kasai Y, Shutsubo K (1999). Petroleum biodegradation in marine environments. J Molec Microbiol Biotechnol.

[40] Yakimov MM, Timmis KN, Golyshin PN (2007). Obligate oil-degrading marine bacteria. Curr Opin Biotechnol.

[41] Rojo F (2009). Degradation of alkanes by bacteria. Environ Microbiol.

[42] McGenity TJ, Folwell BD, McKew BA, Sanni GO (2012). Marine crude-oil biodegradation: a central role for interspecies interactions. Aquat Biosyst.

[43] Schneiker S, Santos VAP, Bartels D, Bekel T, Brecht M, Buhrmester J, Chernikova TN, Denaro R, Ferrer M, Gertler C, Goesmann A, Golyshina OV, Kaminski F, Khachane AN, Lang S, Linke B, AC MH, Meyer F, Nechitaylo T, Pühler A, Regenhardt D, Rupp O, Sabirova JO, Selbitschka W, Yakimov MM, Timmis KN, Vorhölter F, Weidner S, Kaiser O, Golyshin PN (2006). Genome sequence of the ubiquitous hydrocarbon-degrading marine bacterium *Alcanivorax borkumensis*. Nat Biotechnol.

[44] Batista SB, Mounteer A, Amorim FR, Totola MR (2006). Isolation and characterization of biosurfactant/bioemulsifier-producing bacteria from petroleum contaminated sites. Bioresour Technol.

[45] Wang L, Wang W, Lai Q, Shao Z (2010). Gene diversity of CYP153A and AlkB alkane hydroxylases in oil-degrading bacteria isolated from the Atlantic Oceanemi. Environ Microbiol.

[46] Liu Q, Tang J, Bai Z, Hecker M, Giesy JP (2015). Distribution of petroleum degrading genes and factor analysis of petroleum contaminated soil from the Dagang Oilfield, China. Sci Rep.

[47] Wild SR, Jones KC (1995). Polynuclear aromatic hydrocarbons in the united kingdom environment: a preliminary source inventory and budget. Environ Pollut.

