# Supplementary material for: Biogeographical distribution analysis of hydrocarbon degrading and biosurfactant producing genes suggests that near-equatorial biomes have higher abundance of genes with potential for bioremediation
Source: BMC Microbiol. 2017 Jul 27;17:168. doi: 10.1186/s12866-017-1077-4 (PMC5531098; doi:10.1186/s12866-017-1077-4)
Supplement: Supplementary file 1 — Metagenomes Summary. Country, number of samples and sequencing technology for each biome. (DOCX 66 kb) [file 12866_2017_1077_MOESM1_ESM.docx]

Additional file 1: Table S1 - Metagenomes Summary.

| **Biome** | **Country** | **# samples** | **Technology** |
| --- | --- | --- | --- |
| Fracture Water | South Africa | 3 | Illumina |
| Tundra | Canada | 5 | Illumina |
| Tropical Forest | French Guiana | 3 | 454 |
| Temperate Coniferous Forest | Canada | 3 | Illumina |
| Tundra | Russia | 2 | Illumina |
| River Plume | Amazon | 3 | Illumina |
| Saline Desert | India | 3 | Ion Torrent |
| North Atlantic Ocean | Iceland | 1 | Illumina |
| North Atlantic Ocean | Portugal | 5 | Illumina |
| Tropical Atlantic | Brazil | 2 | Illumina |
| Atlantic Forest | Brazil | 1 | Illumina |
| Caatinga | Brazil | 2 | 454 |
| Mangrove | Brazil | 4 | 454 |
| Grassland Soil | USA - Oklahoma | 1 | Illumina |
| Arid Grassland | Australia | 1 | Illumina |
| Temperate Woodland | Australia | 1 | Illumina |
| Temperate Plantation Soil | Australia | 1 | Illumina |
| Adriatic / Ionian Sea | Mediterranium | 2 | Illumina |
| Sea Water | North Pacific | 1 | Illumina |
| Sea Water | South Pacific | 1 | Illumina |
| Sea Water | Indian Ocean | 1 | Illumina |
| Total |  | 46 |  |
