# Supplementary material for: Biogeographical distribution analysis of hydrocarbon degrading and biosurfactant producing genes suggests that near-equatorial biomes have higher abundance of genes with potential for bioremediation
Source: BMC Microbiol. 2017 Jul 27;17:168. doi: 10.1186/s12866-017-1077-4 (PMC5531098; doi:10.1186/s12866-017-1077-4)
Supplement: Supplementary file 2 — Samples Information. Feature, location, run and project SRA information for each sample. (DOCX 120 kb) [file 12866_2017_1077_MOESM2_ESM.docx]

Additional file 5:Table S2 – Metagenomic samples detailed information on SRA project and run.

| WATER | | | | |
| --- | --- | --- | --- | --- |
| **Project** | **Run** | | **Location** | **Feature** |
| SRP039390 | SRR1209976 | | Amazon Continuum Metagenomes | Amazon River Plume |
| SRP039390 | SRR1205251 | | Amazon Continuum Metagenomes | Amazon River Plume |
| SRP039390 | SRR1205253 | | Amazon Continuum Metagenomes | Amazon River Plume |
| SRP039390 | SRR1209977 | | Amazon Continuum Metagenomes | Amazon River Plume |
| ERP009703 | ERR771025 | | Iceland (Faxafloi) | Surface Water |
| ERP009703 | ERR771024 | | Iceland (Faxafloi) | Surface Water |
| ERP009703 | ERR771102 | | Marina Do Funchal (Costa Sul) | Surface Water |
| ERP009703 | ERR771103 | | Quinta Do Lorde (Costa Sul) | Surface Water |
| ERP009703 | ERR770986 | | Vineyard Sound (Ma,Usa) | Surface Water |
| ERP009703 | ERR771096 | | Alcochete | Surface Water |
| SRP049336 | SRR1633226 | | South Africa Thabazimbi | Fracture Water |
| SRP049336 | SRR1633225 | | South Africa: Welkom | Fracture Water |
| SRP049336 | SRR1633224 | | South Africa: Welkom | Fracture Water |
| ERP003708 | ERR599370 | | Trindade And Martin Vaz Islands | Tropical Atlantic Brazil |
| ERP003708 | ERR599347 | | Trindade And Martin Vaz Islands | Tropical Atlantic Brazil |
| ERP003628 | ERR868356 | | Mediterranean Sea | Adriatic Sea - Ionian Sea |
| ERP001736 | ERR599013 | | Indian Ocean | Saline Water |
| ERP001736 | ERR599141 | | Indian Ocean | Saline Water |
| SRP004544 | SRR360932 | | Estuary Of The River Potengi | Brazil Estuary |
| ERP003628 | ERR873961 | | South Pacific Ocean | Saline Water |
| ERP001736 | ERR599093 | | South Pacific Ocean | Saline Water |
| ERP001736 | ERR599079 | | South Pacific Ocean | Saline Water |
| ERP001736 | ERR599042 | | South Pacific Ocean | Saline Water |
| ERP003628 | ERR868385 | | North Pacific Ocean | Saline Water |
| Soil | | | | |
| **Project** | | **Run** | **Location** | **Feature** |
| SRP041239 | | SRR1246239 | Great Rann Of Kutch, Gujarat, India | Saline Desert India |
| SRP041239 | | SRR1246238 | Great Rann Of Kutch, Gujarat, India | Saline Desert India |
| SRP041239 | | SRR1246237 | Great Rann Of Kutch, Gujarat, India | Saline Desert India |
| SRP049520 | | SRR1653578 | Russia - North-East Siberia | Permafrost Alluvial Sediment (Tundra) |
| SRP049520 | | SRR1653579 | Russia - North-East Siberia | Permafrost Alluvial Sediment (Tundra) |
| ERP009498 | | ERR753922 | Canada | Temperate Coniferous Forest |
| ERP009498 | | ERR753927 | Canada | Temperate Coniferous Forest |
| ERP009498 | | ERR753912 | Canada | Temperate Coniferous Forest |
| ERP002426 | | ERR249390 | French Guiana | Forest Soil |
| ERP002426 | | ERR249389 | French Guiana | Forest Soil |
| ERP002426 | | ERR249388 | French Guiana | Forest Soil |
| SRP047512 | | SRR1586250 | Canada: Axel Heiberg Island, Nunavut | Permafrost (Tundra) |
| SRP047512 | | SRR1586314 | Canada: Axel Heiberg Island, Nunavut | Permafrost (Tundra) |
| SRP004544 | | SRR171307 | Ne Brazil - Caatinga Joao Camara Rn | Caatinga |
| SRP004544 | | SRR171684 | Ne Brazil - Caatinga Soil Cariri | Caatinga |
| SRP004544 | | SRR171663 | NE Brazil - Jaguaribe River Mangrove | Mangrove |
| SRP004544 | | SRR171664 | NE Brazil - Timonha River Mangrove | Mangrove |
| SRP004544 | | SRR360810 | NE Brazil - Cocó Mangrove | Mangrove |
| SRP004544 | | SRR360834 | NE Brazil - Pacoti Mangrove | Mangrove |
| ERP008551 | | ERR687888 | Australian - Uluru-Kata Tjuta National Park | Arid Grassland |
| ERP008551 | | ERR687893 | Australian - Conservation And Natural Environments | Temperate Woodland |
| ERP008551 | | ERR687897 | Australian - Dryland Agriculture And Plantations | Temperate Plantation Soil |
