# Supplementary material for: Biogeographical distribution analysis of hydrocarbon degrading and biosurfactant producing genes suggests that near-equatorial biomes have higher abundance of genes with potential for bioremediation
Source: BMC Microbiol. 2017 Jul 27;17:168. doi: 10.1186/s12866-017-1077-4 (PMC5531098; doi:10.1186/s12866-017-1077-4)
Supplement: Supplementary file 3 — Sample Geography. Geographical distribution of the metagenome samples. (DOCX 1078 kb) [file 12866_2017_1077_MOESM3_ESM.docx]

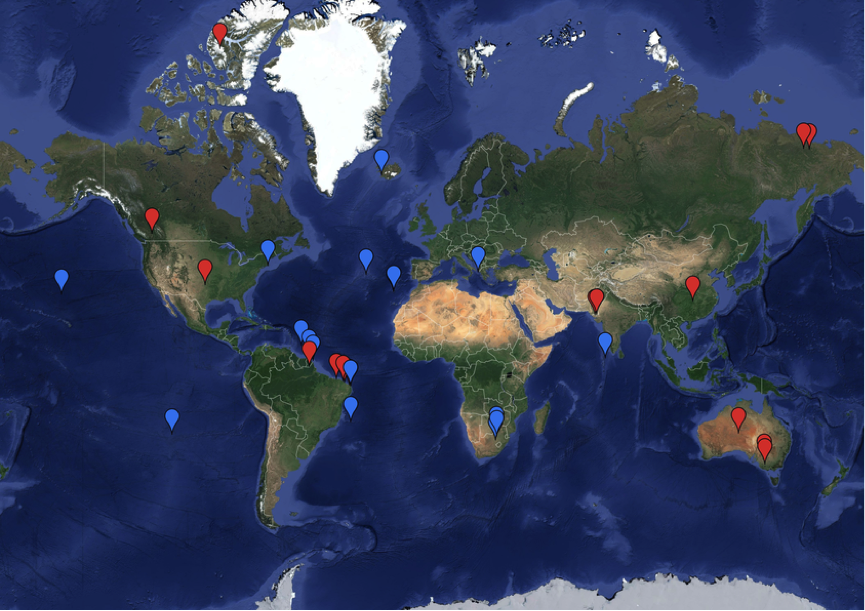


Additional file 2: Figure S1 - Geographical distribution of the metagenome samples. Blue balloons represent water samples and red represent soil samples.
