# Supplementary material for: Biogeographical distribution analysis of hydrocarbon degrading and biosurfactant producing genes suggests that near-equatorial biomes have higher abundance of genes with potential for bioremediation
Source: BMC Microbiol. 2017 Jul 27;17:168. doi: 10.1186/s12866-017-1077-4 (PMC5531098; doi:10.1186/s12866-017-1077-4)
Supplement: Supplementary file 4 — BioSurfDB Pathways. Name and KEGG Map ID for Alkane biodegradation and surfactant biosynthesis pathways analyzed. (DOCX 102 kb) [file 12866_2017_1077_MOESM4_ESM.docx]

Additional file 3: Table S3 - BioSurfDB Analyzed Pathways

| **Alkane Degradation** | **Kegg Entry** | **Surfactants Biosynthesis** | **Kegg Entry** |
| --- | --- | --- | --- |
| Aminobenzoate degradation | [map00627](http://www.genome.jp/dbget-bin/www_bget?pathway:map00627) | Alasan Biosynthesis | N/A |
| Atrazine degradation | [map00791](http://www.genome.jp/dbget-bin/www_bget?pathway:map00791) | Alnumycin Biosynthesis | N/A |
| Benzoate degradation | [map00362](http://www.genome.jp/dbget-bin/www_bget?pathway:map00362) | Amphisin Biosynthesis | N/A |
| Bisphenol degradation | [map00363](http://www.genome.jp/dbget-bin/www_bget?pathway:map00363) | Arthrofactin Biosynthesis | N/A |
| Caprolactam degradation | [map00930](http://www.genome.jp/dbget-bin/www_bget?map00930) | Bacillomycin Biosynthesis | bsu01054 |
| Chloroalkane and chloroalkene degradation | [map00625](http://www.genome.jp/dbget-bin/www_bget?pathway:map00625) | Emulsan Biosynthesis | N/A |
| Chlorocyclohexane and chlorobenzene degradation | [map00361](http://www.genome.jp/dbget-bin/www_bget?map00361) | Generic Surfactant Pathway | N/A |
| Degradation of aromatic compounds | [map01220](http://www.genome.jp/dbget-bin/www_bget?map01220) | Iturin A Biosynthesis | bsu01054 |
| Dioxin degradation | [map00621](http://www.genome.jp/dbget-bin/www_bget?map00621) | Lichenysin Biosynthesis | bsu01054 |
| Drug metabolism - cytochrome P450 | [map00982](http://www.genome.jp/dbget-bin/www_bget?pathway:map00982) | Lipopeptide Surfactants Biosynthesis | N/A |
| Ethylbenzene degradation | [map00642](http://www.genome.jp/dbget-bin/www_bget?pathway:map00642) | Nonribosomal peptide structures | map01054 |
| Fatty acid degradation (alkane degradation) | [map00071](http://www.genome.jp/dbget-bin/www_bget?pathway:map00071) | PPAR signaling pathway | ko03320 |
| Fluorobenzoate degradation | [map00364](http://www.genome.jp/dbget-bin/www_bget?pathway:map00364) | Phenylpropanoid biosynthesis | map00940 |
| Metabolism of xenobiotics by cytochrome P450 | [map00980](http://www.genome.jp/dbget-bin/www_bget?pathway:map00980) | Phosphatidylethanolamine Biosynthesis | map00564 |
| Naphthalene degradation | [map00626](http://www.genome.jp/dbget-bin/www_bget?pathway:map00626) | Plipastatin Biosynthesis | bsu01054 |
| Nitrotoluene degradation | [map00633](http://www.genome.jp/dbget-bin/www_bget?pathway:map00633) | Polyketide sugar unit biosynthesis | map00523 |
| Polycyclic aromatic hydrocarbon degradation | [map00624](http://www.genome.jp/dbget-bin/www_bget?pathway:map00624) | Putisolvins Biosynthesis | N/A |
| Toluene degradation | [map00623](http://www.genome.jp/dbget-bin/www_bget?pathway:map00623) | Rhamnolipid Biosynthesis | N/A |
| Xylene degradation | [map00622](http://www.genome.jp/dbget-bin/www_bget?pathway:map00622) | Serrawettin Biosynthesis | N/A |
| Styrene degradation | map00643 | Streptomycin biosynthesis | [map00521](http://www.genome.jp/dbget-bin/www_bget?pathway:map00521) |
|  |  | Surfactin Biosynthesis | bsu01054 |
|  |  | Trehalolipids Biosynthesis | N/A |
