# Supplementary material for: Biogeographical distribution analysis of hydrocarbon degrading and biosurfactant producing genes suggests that near-equatorial biomes have higher abundance of genes with potential for bioremediation
Source: BMC Microbiol. 2017 Jul 27;17:168. doi: 10.1186/s12866-017-1077-4 (PMC5531098; doi:10.1186/s12866-017-1077-4)
Supplement: Supplementary file 5 — Rarefaction Curve. Rarefaction Curves performed in MEGAN. (DOCX 2185 kb) [file 12866_2017_1077_MOESM5_ESM.docx]

*
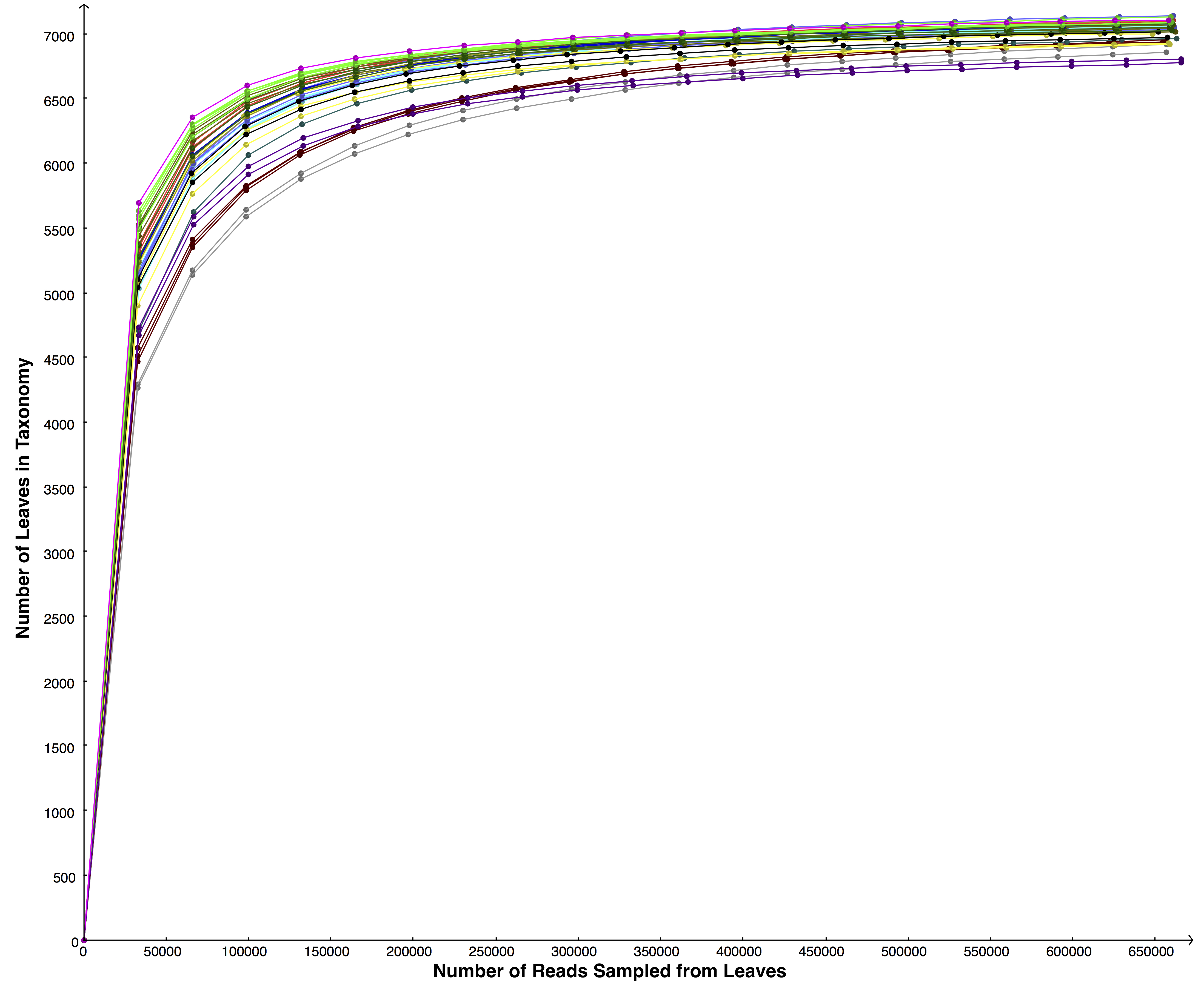
*

Additional file 4: Figure S2 - Rarefaction Curves performed in MEGAN, representing the number of leaves in taxonomy, depending on the number of reads sampled from leaves. All the curves reach a plateau, suggesting that the number of reads is enough to cover the majority of the taxons.
